# 118 SNPs of folate-related genes and risks of spina bifida and conotruncal heart defects

**DOI:** 10.1186/1471-2350-10-49

**Published:** 2009-06-03

**Authors:** Gary M Shaw, Wei Lu, Huiping Zhu, Wei Yang, Farren BS Briggs, Suzan L Carmichael, Lisa F Barcellos, Edward J Lammer, Richard H Finnell

**Affiliations:** 1 Department of Pediatrics, Division of Neonatal & Developmental Medicine, Stanford University School of Medicine, Stanford, CA, USA; 2 Institute of Biosciences and Technology, Texas A&M Health Science Center, Houston, TX, USA; 3 California Research Division, March of Dimes, California Research Division, Oakland, CA, USA; 4 School of Public Health, University of California, Berkeley, School of Public Health, Berkeley, CA, USA; 5 Children's Hospital Oakland Research Institute, Oakland, CA, USA

## Abstract

**Background:**

Folic acid taken in early pregnancy reduces risks for delivering offspring with several congenital anomalies. The mechanism by which folic acid reduces risk is unknown. Investigations into genetic variation that influences transport and metabolism of folate will help fill this data gap. We focused on 118 SNPs involved in folate transport and metabolism.

**Methods:**

Using data from a California population-based registry, we investigated whether risks of spina bifida or conotruncal heart defects were influenced by 118 single nucleotide polymorphisms (SNPs) associated with the complex folate pathway. This case-control study included 259 infants with spina bifida and a random sample of 359 nonmalformed control infants born during 1983–86 or 1994–95. It also included 214 infants with conotruncal heart defects born during 1983–86. Infant genotyping was performed blinded to case or control status using a designed SNPlex assay. We examined single SNP effects for each of the 118 SNPs, as well as haplotypes, for each of the two outcomes.

**Results:**

Few odds ratios (ORs) revealed sizable departures from 1.0. With respect to spina bifida, we observed ORs with 95% confidence intervals that did not include 1.0 for the following SNPs (heterozygous or homozygous) relative to the reference genotype: *BHMT *(rs3733890) OR = 1.8 (1.1–3.1), *CBS *(rs2851391) OR = 2.0 (1.2–3.1); *CBS *(rs234713) OR = 2.9 (1.3–6.7); *MTHFD1 *(rs2236224) OR = 1.7 (1.1–2.7); *MTHFD1 *(hcv11462908) OR = 0.2 (0–0.9); *MTHFD2 *(rs702465) OR = 0.6 (0.4–0.9); *MTHFD2 *(rs7571842) OR = 0.6 (0.4–0.9); *MTHFR *(rs1801133) OR = 2.0 (1.2–3.1); *MTRR *(rs162036) OR = 3.0 (1.5–5.9); *MTRR *(rs10380) OR = 3.4 (1.6–7.1); *MTRR *(rs1801394) OR = 0.7 (0.5–0.9); *MTRR *(rs9332) OR = 2.7 (1.3–5.3); *TYMS *(rs2847149) OR = 2.2 (1.4–3.5); *TYMS *(rs1001761) OR = 2.4 (1.5–3.8); and *TYMS *(rs502396) OR = 2.1 (1.3–3.3). However, multiple SNPs observed for a given gene showed evidence of linkage disequilibrium indicating that the observed SNPs were not individually contributing to risk. We did not observe any ORs with confidence intervals that did not include 1.0 for any of the studied SNPs with conotruncal heart defects. Haplotype reconstruction showed statistical evidence of nonrandom associations with *TYMS*, *MTHFR*, *BHMT *and *MTR *for spina bifida.

**Conclusion:**

Our observations do not implicate a particular folate transport or metabolism gene to be strongly associated with risks for spina bifida or conotruncal defects.

## Background

Periconceptional vitamin supplementation with folic acid substantially reduces risks of women having neural tube defect-affected pregnancies [1,2] and has been implicated in reducing risks of several other congenital anomalies, including orofacial clefts and selected heart defects [3-11]. Mechanisms underlying these reduced risks have not been elucidated, although it has been speculated that supplementation with vitamins containing folic acid restores some normal developmental function that is genetically compromised in selected infants.

Investigating genetic variation that influences cellular absorption, transport, and metabolism of folate may offer insight into this unknown developmentally protective mechanism. Indeed, numerous investigations of genes that are specifically involved with folate metabolism have yielded at least one gene, 5, 10-methylenetetrahydrofolate reductase (*MTHFR*), that has been associated with a modest increased risk of neural tube defects (e.g., [12-17]), and possibly heart defects [18,19]. Observed risks with the two principal *MTHFR *variants, however, do not appear to account for a large proportion of the etiologic fraction of any of these defects, under the assumption that *MTHFR *variants have a causal role [17]. Thus, further investigation of other folate-related genes is necessary to reveal clues about mechanisms underlying the potential embryonic protective effects of folic acid supplementation.

We hypothesized that genetic susceptibility of fetal metabolism or transport of folate puts fetuses at risk for selected congenital anomalies. Using population-based data, we investigated 118 single nucleotide polymorphisms (SNPs) in 14 genes in the complex folate pathway as risk factors for spina bifida and conotruncal heart defects.

## Methods

This population-based case-control study included infants with spina bifida or conotruncal heart defects diagnosed within 1 year after birth among infants and fetal deaths delivered to women residing in most California counties. Data were derived from the California Birth Defects Monitoring Program [20], a population-based active surveillance system for collecting information on infants and fetuses with congenital malformations. Diagnostic and demographic information was collected by program staff from multiple sources of medical records for all liveborn and stillborn fetuses (defined as >20 weeks gestation). Overall ascertainment for major malformations has been estimated as 97% complete [21]. Eligible were live born infants only because the source of DNA was from newborn screening cards.

Included were 259 infants with spina bifida and a random sample of 359 nonmalformed control infants born during 1983–86 and 1994–95 in selected counties in California. Also included for study were 214 infants with conotruncal heart defects, specifically d-transposition of the great arteries and tetralogy of Fallot. The random sample of 1983–86 controls for conotruncal heart defects included 220 of the overall 359. Newborn bloodspots were obtained from the State of California and their use in this study was consistent with the consent procedures at the time of sample collection. The protocol for this study was reviewed and approved by the State of California Health and Welfare Agency Committee for the Protection of Human Subjects.

Genomic DNA was extracted from dried blood spots on filter paper using the Puregene DNA Extraction Kit (Gentra, Minneapolis, MN). Prior to genotyping, genomic DNA was amplified using a commercial multiple displacement amplification (MDA) kit, GenomePhi (GE Healthcare, Piscataway, NJ). The MDA method relies on isothermal amplification using the DNA polymerase of the bacteriophage phi29 and is a recently developed technique for high performance WGA. MDA has been demonstrated to be reliable for genotyping, with the most favorable call rates, best genomic coverage, and lowest amplification bias [22]. Studies indicate no discernable difference between WGA samples with GenomiPhi kit and the original DNA templates [23,24]. The whole genome amplification (WGA) product was then quantified using RNase P method (AppliedBiosystems, Foster City, CA). 150 ng WGA product was then used for each SNPlex assay pool which contained about 48 SNPs.

Genotype analyses were performed using SNPlex assays (AppliedBiosystems, Foster City, CA). SNP markers were selected using the SNPBrowser™ program (version 3.0) provided by AppliedBiosystems Inc. This program allowed selection of SNP markers from the HapMap database. For each target gene, tagging SNPs were selected based on the pairwise r^2 ^> = 0.8. SNPs with minor allele frequencies lower than 10% in Caucasians were excluded. All validated non-synonymous SNPs were included. Successful rates for SNPlex assays were >96% for 75 SNPs, from 90% to 96% for 32 SNPs, from 70% to 90% for 7 SNPs. 15 SNPs suffered from more than 30% failure rates. In a subsequent effort to fill in the missing genotyping data and obtain higher call rate, we performed TaqMan SNP assays (Appliedbiosystems, Foster City, CA) for 22 of these SNPs on an ABI 7900 Genetic Analyzer.

All genotyping was performed blinded to subject's case or control status. Case and control infants were genotyped for 129 SNPs. Failure to obtain unambiguous genotype data on >50% of the samples for 11 SNPs (*CBS *rs1801181 and rs12329790; *MTHFR *rs1537514 and rs7533315; *MTR *rs10925257, *NOS3 *rs1800780 and hcv11631000; *RFC1 *rs1051266, rs4819130, hcv16186310, and rs7278825) resulted in their elimination from further analyses. The remaining 118 SNPs are shown in Table 1. The percentage of control study subjects (percentages were similar for cases) for whom genotype could be assigned is also shown in Table 1.

**Table 1:** Fourteen folate-related genes and 118 SNPs

**Gene**	**Change**	**Chromosome**	**Base Position**	**SNP_ID**	**Type/Comment**	**Percent Genotyped^1^**
*BHMT*	R (A/G)	5	78457715	rs3733890	exon, nonsynonymous R239Q	100
*BHMT*	Y (C/T)	5	78471967	rs1915706	Intergenic/Unknown	96.4
*BHMT*	(G/C)	5	78567093	rs1316753	Tag, BHMT	100
*BHMT*	M (C/A)	5	78465350	rs617219	intergenic	96.4
*BHMT*	M (A/C)	5	78438303	rs645112	Intergenic/Unknown	96.9
*BHMT*	W (A/T)	5	78462964	rs585800	untranslated region	94.2
*BHMT*	S (C/G)	5	78559288	rs3829809	Tag, BHMT	100
*BHMT*	Y (C/T)	5	78452172	rs567754	intron	95.8
*BHMT2*	M (A/C)	5	78400443	rs642431	intergenic-BHMT2;intron-DMGDH	91.1
*BHMT2*	R (A/G)	5	78405657	rs626105	intron	96.1
*BHMT2*	Y (C/T)	5	78409187	rs682985	exon, synonymous	95.5
*BHMT2*	M (A/C)	5	78387392	rs2253262	exon, synonymous	96.4
*BHMT2*	K(G/T)	5	78402082	rs670220	Validated	96.7
*BHMT2*	R (A/G)	5	78404048	rs592052	intron	99.2
*BHMT2*	R (A/G)	5	78419219	rs597560	intron	98.3
*CBS*	Y (T/C)	21	43360473	rs2851391	intron	92.5
*CBS*	R (A/G)	21	43359173	rs2298759	intron	72.4
*CBS*	Y (T/C)	21	43361102	rs234714	intron	90
*CBS*	S (C/G)	21	43346936	rs1051319	untranslated region	91.9
*CBS*	Y (T/C)	21	43376503	rs234784	Tag, CBS	99.7
*CBS*	N (A/C/G/T)	21	43346760	rs12613	untranslated region	92.5
*CBS*	S (C/G)	21	43377074	rs234785	Tag, CBS	100
*CBS*	R (A/G)	21	43360960	rs234713	intron	91.1
*CBS*	Y (C/T)	21	43376312	rs234783	Tag, CBS	100
*DHFR*	Y(C/T)	5	79986537	rs1650697	Validated nsSNP	92.2
*DHFR*	W(A/T)	5	79957572	rs12109877	Validated	94.2
*DHFR*	Y(C/T)	5	79987790	rs380691	Validated	95.5
*DHFR*	M(A/C)	5	79985331	rs1478834	Validated	96.4
*DHFR*	Y(C/T)	5	79966012	rs1643638	Validated	92.8
*DHFR*	M(A/C)	5	79961366	rs2618372	Validated	96.9
*DHFR*	R (A/G)	5	79980489	rs13161245	Validated	96.1
*DHFR*	Y(C/T)	5	79975899	rs1643650	Validated	94.7
*DHFR*	K(G/T)	5	79981467	rs836821	Validated	97.5
*FOLR1*	Y (C/T)	11	73373406	rs1540087	untranslated region	95.8
*FOLR1*	W (T/A)	11	73380857	rs11235462	Tag, FOLR1	100
*FOLR1*	R (A/G)	11	73372879	rs2071010	untranslated region	91.9
*FOLR2*	R (A/G)	11	73404256	rs2298444	intron	92.2
*FOLR2*	R (A/G)	11	73402049	rs514933	intron	100
*FOLR2*	W (A/T)	11	73401368	rs651646	untranslated region	100
*MTHFD1*	Y (C/T)	14	63984935	rs2236222	intron	95.5
*MTHFD1*	Y (C/T)	14	63978904	rs2236224	intron	97.8
*MTHFD1*	Y (C/T)	14	63952133	rs1950902	exon, nonsynonymous	90.5
*MTHFD1*	Y (C/T)	14	63978598	rs2236225	exon, nonsynonymous G1958A (R653Q)	100
*MTHFD1*	(T/A)	14	63999040	hCV11462908	Tag, MTHFD1	100
*MTHFD1*	R (A/G)	14	63957808	hCV11660794	intron	95.3
*MTHFD1*	R (A/G)	14	63988165	rs11849530	intron	95.8
*MTHFD1*	R (A/G)	14	63990418	rs1256146	intron	95
*MTHFD1*	Y (C/T)	14	63985918	rs10137921	exon, nonsynonymous	96.4
*MTHFD1*	Y (C/T)	14	63980547	rs1256142	intron	97.8
*MTHFD2*	Y (T/C)	2	74304595	rs11126426	Intergenic, Tag	100
*MTHFD2*	(T/A)	2	74280806	rs702465	Intergenic, Tag	96.7
*MTHFD2*	R (A/G)	2	74313429	rs1667599	Intergenic, Tag	100
*MTHFD2*	R (A/G)	2	74340847	rs1667627	Validated	96.1
*MTHFD2*	W (A/T)	2	74333849	rs828858	Intergenic, Tag	100
*MTHFD2*	(C/G)	2	74281605	rs702466	Intergenic, Tag	99.7
*MTHFD2*	R (A/G)	2	74372559	rs7571842	Intergenic, Tag	100
*MTHFD2*	R (A/G)	2	74348376	rs828903	Validated	94.4
*MTHFR*	R (A/G)	1	11801310	rs3737964	Validated	95.8
*MTHFR*	R (A/G)	1	11823734	rs535107	Intergenic, Tag	93.3
*MTHFR*	K(G/T)	1	11798240	rs1931226	Validated	96.9
*MTHFR*	R(A/G)	1	11780518	rs4846048	Validated	89.7
*MTHFR*	Y (C/T)	1	11796598	rs7525338	Validated	97.5
*MTHFR*	R (A/G)	1	11785193	rs2274976	exon, nonsynonymous	93
*MTHFR*	Y (C/T)	1	11792217	rs4846052	intron	96.9
*MTHFR*	Y (C/T)	1	11790644	rs1801133	exon, nonsynonymous C677T	99.4
*MTHFR*	R (A/G)	1	11775209	rs1889292	Intergenic, Tag	100
*MTHFR*	Y (C/T)	1	11797323	rs2066470	exon, synonymous	95.3
*MTHFR*	R (A/G)	1	11788723	rs4846051	exon, synonymous	93
*MTHFR*	R (A/G)	1	11786566	rs1476413	intron	93.9
*MTHFR*	M (A/C)	1	11788742	rs1801131	exon, nonsynonymous A1298C	99.7
*MTR*	M (A/C)	1	233374717	rs2275565	Validated	96.1
*MTR*	Y (C/T)	1	233322616	rs1806505	intron	97.5
*MTR*	K(G/T)	1	233386474	rs3820571	Validated	96.1
*MTR*	Y (C/T)	1	233335898	rs3754255	Validated	94.7
*MTR*	S(C/G)	1	233381346	rs10802569	Validated	96.1
*MTR*	R (A/G)	1	233376992	rs1266164	intron	96.1
*MTR*	R (A/G)	1	233374541	rs1805087	exon, nonsynonymous A2756G	96.4
*MTR*	W(A/T)	1	233385428	rs4659743	Validated	98.3
*MTR*	K(G/T)	1	233390667	rs6676866	Validated	98.3
*MTR*	S(C/G)	1	233315110	rs12060570	Validated	98.6
*MTR*	W(A/T)	1	233306545	rs955516	Validated	99.2
*MTR*	K (G/T)	1	233313831	rs4077829	intron	96.7
*MTR*	R (A/G)	1	233364202	rs1770449	intron	94.4
*MTR*	S (C/G)	1	233353709	rs3768139	intron	95.5
*MTR*	R (A/G)	1	233300165	rs4659724	intron	97.2
*MTR*	Y (C/T)	1	233327367	rs6668344	intron	96.4
*MTR*	R (A/G)	1	233367345	rs7367859	Validated	93.9
*MTR*	K (G/T)	1	233354605	rs3768142	Validated	96.1
*MTR*	Y (C/T)	1	233348403	rs10925252	Validated	96.9
*MTR*	R (A/G)	1	233380610	rs2229276	exon, synonymous	95
*MTR*	R (A/G)	1	233388346	rs1050993	untranslated region	97.2
*MTRR*	R (A/G)	5	7938959	rs162036	Validated nsSNP Lys/Arg	95.5
*MTRR*	S (C/G)	5	7944506	rs16879334	exon, nonsynonymous Pro/Arg	90
*MTRR*	R (G/A)	5	7950319	rs1802059	exon, synonymous	94.7
*MTRR*	R (G/A)	5	7942216	rs2287779	exon, synonymous	97.2
*MTRR*	R (A/G)	5	7927847	rs326120	intron	87.7
*MTRR*	Y (C/T)	5	7950191	rs10380	exon, nonsynonymous, His/Tyr	96.4
*MTRR*	R (A/G)	5	7923973	rs1801394	exon, nonsynonymous	96.7
*MTRR*	Y (C/T)	5	7953712	rs9332	UTR	92.2
*MTRR*	S (C/G)	5	7938907	rs10064631	exon, nonsynonymous	95
*MTRR*	W (A/T)	5	7931424	rs2303080	exon, nonsynonymous	96.1
*MTRR*	R (A/G)	5	7949511	rs3776455	intron	95
*MTRR*	R (A/G)	5	7931179	rs1532268	exon, nonsynonymous	95
*MTRR*	R (A/G)	5	7945310	rs162048	intron	98.6
*NOS3*	R (A/G)	7	150145737	rs891512	intron	86.6
*NOS3*	R (A/G)	7	150127591	rs1800779	untranslated region	87.5
*NOS3*	Y (C/T)	7	150148555	rs3918211	exon, synonymous	96.9
*RFC1*	K (G/T)	21	45761011	rs3788189	intron	81.1
*RFC1*	R (A/G)	21	45755537	rs12483377	Tag, RFC	100
*RFC1*	R (A/G)	21	45756112	rs2236484	Intron, Tag	98.6
*RFC1*	R (A/G)	21	45761386	rs3788190	Intron, Tag	91.1
*RFC1*	S (C/G)	21	45750430	rs10483080	intron	99.7
*RFC1*	Y (C/T)	21	45777720	rs2330183	intron	91.4
*TYMS*	Y (C/T)	18	652215	rs11540152	exon, nonsynonymous	95.8
*TYMS*	Y (C/T)	18	660414	rs2853532	intron	96.4
*TYMS*	R (A/G)	18	656371	rs2847149	intron	97.2
*TYMS*	Y (C/T)	18	652103	rs1001761	intron	98.9
*TYMS*	Y (C/T)	18	649236	rs502396	intron	97.8

^1^Percent of 359 controls genotyped for each SNP.

Abbreviations: *BHMT *= betaine homocysteine methyltransferase; *BHMT2 *betaine homocysteine methyltransferase-2; *CBS *= cystathione beta synthase; *DHFR *= dihydrofolate reductase; *FOLR1 *folate receptor 1; *FOLR2 *folate receptor 2; *MTHFD1 *= methylenetetrahydrofolate dehydrogenase 1; *MTHFD2 *= methylenetetrahydrofolate dehydrogenase 2; *MTHFR *= methylenetetrahydrofolate reductase; *MTR *= methionine synthase; *MTRR *= methionine synthase reductase; *NOS3 *= nitric oxide synthase; *RFC1 *= reduced folate carrier 1; *TYMS *= thymidylate synthase.

Genotypes among controls were analyzed to verify that their distributions fit Hardy-Weinberg expectations. Genotypes for each SNP were statistically consistent with Hardy-Weinberg expectations. Odds ratios and 95% confidence intervals (CI) were used to estimate risks. These measures were calculated using SAS software (version 9.1). Information on maternal race/ethnicity was obtained for case and control infants from California birth certificates. Logistic regression was used to compute risk estimates adjusted for maternal race/ethnicity (white Hispanic; white nonHispanic, and other). Analyses estimated defect risks (spina bifida or conotruncal heart defects) for each SNP assuming a recessive model, i.e., homozygous variant genotype compared to homozygous reference genotype and heterozygous variant genotype compared to homozygous reference genotype. In addition to single SNP-at-a-time analyses, we explored haplotype block analyses. Haplotype analyses were performed using Haploview version 3.32. Identified blocks were assessed with odds ratios.

## Results

Numbers of case and control infants stratified by race/ethnicity are shown in Table 2. These data show the expected greater frequency of Hispanics in the spina bifida case group.

**Table 2:** Racial/ethnic percentages of malformed cases and non-malformed controls, California 1983–86 and 1994–95.

	**Spina Bifida**		**Conotruncal Heart**	
	Cases n = 259 %^2^	Controls n = 359 %^2^	Cases n = 214 %^2^	Controls n = 220^1^ %^2^
				
**Race/Ethnicity**				
White, Hispanic	50.6	31.5	17.8	18.6
White, nonHispanic	35.9	47.4	53.3	61.8
Other	12.0	20.6	26.2	18.6

^1^The number of controls born in the period 1983–86 among the 359 selected for the overall study period 1983–86 and 1994–95. The 220 represent the birth years of cases with conotruncal heart defects.

^2^Percentages may not equal 100 owing to missing data or rounding.

We examined risks for each of the 118 SNPs and for each of the two birth defect outcome (Additional file 1). Few odds ratios (ORs) revealed sizable departures from 1.0. Given the large number of comparisons (n = 472) we expected more ORs to be substantially different from 1.0 by chance. With respect to spina bifida, we observed ORs with confidence intervals that did not include 1.0 for the following SNPs (heterozygous or homozygous) relative to the reference genotype: *BHMT *(rs3733890) OR = 1.8 (1.1–3.1), *CBS *(rs2851391) OR = 2.0 (1.2–3.1); *CBS *(rs234713) OR = 2.9 (1.3–6.7); *MTHFD1 *(rs2236224) OR = 1.7 (1.1–2.7); *MTHFD1 *(hcv11462908) OR = 0.2 (0–0.9); *MTHFD2 *(rs702465) OR = 0.6 (0.4–0.9); *MTHFD2 *(rs7571842) OR = 0.6 (0.4–0.9); *MTHFR *(rs1801133) OR = 2.0 (1.2–3.1); *MTRR *(rs162036) OR = 3.0 (1.5–5.9); *MTRR *(rs10380) OR = 3.4 (1.6–7.1); *MTRR *(rs1801394) OR = 0.7 (0.5–0.9); *MTRR *(rs9332) OR = 2.7 (1.3–5.3); *TYMS *(rs2847149) OR = 2.2 (1.4–3.5); *TYMS *(rs1001761) OR = 2.4 (1.5–3.8); and *TYMS *(rs502396) OR = 2.1 (1.3–3.3). Each gene involving multiple SNP associations was investigated for linkage disequilibrium. Modest to strong evidence for linkage disequilibrium was observed for SNPs in each gene, i.e., D' ranged from 0.44 to 1.0 with all p values < 10^-4^. With respect to conotruncal heart defects, we did not observe any OR with a confidence interval that did not include 1.0.

We did not observe evidence to indicate that risk patterns were confounded by race/ethnicity groupings, i.e., observed ORs were not substantially altered after adjusting for maternal race/ethnicity (not shown, available from authors upon request).

Haplotypes, reconstructed for each gene based on studied SNPs, were explored to assess risks for each case group. A total of 77 of the 118 studied SNPs formed 17 haplotype blocks. As shown in Table 3, blocks for *TYMS*, *MTHFR*, *BHMT*, and *MTR *showed some evidence of nonrandom effects for spina bifida. For each of these haplotypes we observed decreased risk associated with the lower frequency haplotype relative to the most frequent haplotype. Similar to SNP analyses, haplotype analyses for conotruncal heart defects did not reveal evidence of nonrandom effects, with the exception of one haplotype block for *MTR *(Table 4).

**Table 3:** Haplotype associations with risks of spina bifida

**Haplotype Block**	**Frequency**	**Odds Ratio (95% CI)**
***TYMS***		
CGC	0.500	REF
TAT	0.373	0.7 (0.6–0.9)
TAC	0.115	0.5 (0.3–0.7)
***MTRR***		
ATTAGCAACAC	0.264	REF
ACTGGCAGTGT	0.213	1.4 (1.0–1.9)
ACTAGCAACGC	0.201	0.8 (0.6–1.1)
GCTAGCGGCGC	0.162	1.1 (0.7–1.5)
ACAAAGAGCGC	0.055	1.1 (0.7–1.9)
ACTAGCAGCGC	0.034	0.6 (0.3–1.3)
ACTAAGAGCGC	0.027	1.2 (0.6–2.6)
ACTGGCAGCGT	0.011	1.4 (0.5–4.1)
MTHFR*		
GGG	0.656	REF
AGA	0.163	0.9 (0.6–1.2)
AGG	0.121	0.9 (0.6–1.2)
AAA	0.057	0.6 (0.3–1.0)
MTHFR**		
TCCCA	0.368	REF
CCCCA	0.231	0.7 (0.5–0.9)
CTCTG	0.180	0.8 (0.6–1.1)
CTTCG	0.099	0.6 (0.4–0.9)
CTCCG	0.063	0.7 (0.5–1.2)
CTCCA	0.037	1.0 (0.5–1.8)
***CBS***		
CG	0.889	REF
TC	0.055	1.2 (0.7–1.9)
CC	0.053	0.6 (0.3–1.0)
RFC1*		
CG	0.856	REF
GG	0.079	1.1 (0.7–1.7)
GA	0.063	1.0 (0.6–1.6)
RFC1**		
TG	0.486	REF
GA	0.463	0.9 (0.7–1.2)
GG	0.046	0.6 (0.3–1.0)
MTHFD1*		
CT	0.486	REF
TC	0.429	1.3 (1.0–1.6)
CC	0.080	0.9 (0.6–1.4)
MTHFD1**		
GT	0.825	REF
AA	0.167	0.9 (0.6–1.2)
***FOLR2***		
TA	0.549	REF
AG	0.356	1.0 (0.8–1.3)
AA	0.093	1.0 (0.7–1.6)
MTHFD2*		
TA	0.589	REF
CA	0.321	1.1 (0.9–1.4)
CG	0.089	1.1 (0.7–1.6)
MTHFD2**		
TC	0.388	REF
TT	0.332	1.2 (0.9–1.5)
AT	0.276	1.0 (0.8–1.4)
***BHMT2***		
GGGTCA	0.466	REF
TAACTC	0.219	1.0 (0.7–1.3)
GAGCTC	0.171	1.1 (0.8–1.6)
GAGTCA	0.091	1.0 (0.6–1.5)
GGGTCC	0.022	0.7 (0.3–1.7)
BHMT*		
CAA	0.339	REF
TGA	0.326	0.7 (0.5–0.9)
CGT	0.172	0.7 (0.5–1.0)
CGA	0.158	0.9 (0.6–1.2)
BHMT**		
AC	0.501	REF
CT	0.373	0.8 (0.7–1.1)
AT	0.120	0.9 (0.6–1.3)
***DHFR***		
CTTACCA	0.402	REF
CTTACCG	0.390	0.9 (0.7–1.2)
ACCGAAA	0.201	0.9 (0.7–1.3)
***MTR***		
AATCTTTCCTAGAGGGCTTGG	0.373	REF
GTGGCCCTGGGAAGAAGAGAT	0.262	1.0 (0.7–1.3)
GTGGCCCTCTAGGTGACTTGG	0.190	0.9 (0.7–1.3)
GTGGCCCTGGGGAGAAGAGAT	0.045	1.4 (0.8–2.5)
GTGGCCTTCTAGATGACTTGT	0.040	0.6 (0.3–1.2)
GTGGCCCTCGAAAGGAGTTGT	0.032	0.3 (0.1–0.6)

***TYMS ***included rs1001761, rs2847149 and, rs2853532; ***MTRR ***included rs326120, rs1532268, rs2303080, rs162036, rs2287779, rs16879334, rs162048, rs3776455, rs10380

rs1802059, and rs9332; MTHFR* included rs1889292, rs2274976, and rs1476413; MTHFR** included rs1801133, rs4846052, rs2066470, rs3737964, and rs535107; ***CBS ***included rs12613 and rs 1051319; RFC1* included rs10483080 and rs12483377; RFC1** included rs3788189 and rs3788190; MTHFD1* included rs2236224 and rs1256142; MTHFD1** included rs1256146 and hcv11462908; ***FOLR2 ***included rs651646 and rs514933; MTHFD2* included rs11126426 and rs1667599; MTHFD2** included rs828858 and rs1667627; ***BHMT2 ***included rs670220, rs592052, rs626105, rs682985, rs597560, and rs645112; BHMT* included rs567754, rs3733890, and rs585800; BHMT** included rs617219 and rs1915706; ***DHFR ***included rs2618372, rs1643638, rs1643650, rs13161245, rs836821, rs1478834, and rs380691; ***MTR ***included rs4659724, rs955516, rs4077829, rs12060570, rs1806505, rs6668344, rs3754255, rs10925252, rs3768139, rs3768142, rs1770449, rs7367859, rs1805087, rs2275565, rs1266164, rs2229276, rs10802569, rs4659743, rs3820571, rs1050993, and rs6676866.

**Table 4:** Haplotype association with risks of conotruncal heart defects

**Haplotype**	**Frequency**	**Odds Ratios (95% CI)**
**Block 19 (*MTR*)**		
AATCTTTCCTAGAGGGCTTGG	0.354	REF
GTGGCCCTGGGAAGAAGAGAT	0.272	1.1 (0.7–1.5)
GTGGCCCTCTAGGTGACTTGG	0.189	1.2 (0.8–1.8)
GTGGCCTTCTAGATGACTTGT	0.048	1.5 (0.8–3.0)
GTGGCCCTCGAAAGGAGTTGT	0.035	1.0 (0.5–2.1)
GTGGCCCTGGGGAGAAGAGAT	0.021	0.9 (0.3–2.2)
GATCTTTCCTAGAGGGCTTGG	0.013	10.7 (1.4–84.8)

Block 19 included rs4659724, rs955516, rs4077829, rs12060570, rs1806505, rs6668344, rs3754255, rs10925252, rs3768139, rs3768142, rs1770449, rs7367859, rs1805087, rs2275565, rs1266164, rs2229276, rs10802569, rs4659743, rs3820571, rs1050993, and

rs6676866.

Haplotype analyses were stratified by race/ethnic background (Hispanic white and nonHispanic white). We observed evidence of a nonrandom haplotype association with *TYMS *for spina bifida and conotruncal heart defects among nonHispanic whites. Lack of evidence for other haplotypes that were observed overall was likely the result of smaller sample sizes from stratification.

## Discussion

In this California population we found only modest evidence that polymorphisms in 14 folate-related genes contributed to risk of spina bifida. SNPs contributing risks were in *BHMT*, *CBS*, *MTHFD1*, *MTHFD2*, *MTHFR*, *MTRR*, and *TYMS*. Haplotype association analyses further identified *TYMS *and *MTHFR *as potential contributors to spina bifida risk. In general, however, most of these folate-related genes showed little evidence for a gene-only effect on risk of spina bifida, and even less, on risks of conotruncal heart defects.

The 14 genes studied here have been implicated in the complex metabolic cycle involving folate (e.g., [25-27]). To our knowledge, this study contained the largest number of SNPs in folate-related genes interrogated as risk factors for human spina bifida or conotruncal heart defects. Previous studies have included some of the SNPs examined here. For example, Boyles and colleagues [28] studied 28 SNPs in 11 folate-related genes and found that only *BHMT *(rs3733890) was associated with increased spina bifida risk. This *BHMT *association is consistent with our findings that showed an odds ratio of 1.8 (1.1–3.1).

Many studies have explored *MTHFR *677 (rs1801133) polymorphism. A range of risks, including no-effect, has been reported for this SNP relative to spina bifida. Botto and Yang [15] in a meta-analysis demonstrated a pooled odds ratio of 1.8 for spina bifida among infants homozygous for 677T. A few studies have also explored this 677 SNP in *MTHFR *as a risk factor for selected congenital heart defects, with most investigations finding no or little association [18,19,29-31]. We did observe a 2-fold increased risk of spina bifida associated with this SNP for homozygous infants. Further, haplotype analyses showed some association for the *MTHFR *gene as well.

Methionine synthase (MTR) is a vitamin B_12 _dependent enzyme that is essential for the remethylation of homocysteine to methionine. The enzyme is required by cells for the essential accumulation of folate [32]. One particular SNP (A2756G; rs1805087) has been considerably investigated, with increased risks of NTDs reported in some studies [33-35], but not in others [36,37]. We did not find an increased risk for spina bifida or conotruncal heart defects associated with this SNP or any other SNP of *MTR*.

Cystathione beta synthase (CBS) is critical to the degradation of homocysteine to cysteine. Regulation of this pyridoxal phosphate-dependent enzyme catalyzes the hydroxyl group of serine with the thiolate of homocysteine [38]. The polymorphism in the *CBS *gene that has received the most study is a 68 bp insertion (844ins68), with predominantly no associations observed for NTDs [27]. This polymorphism was not investigated in the current study. We did observe, however, two *CBS *SNPs (rs2851391 and rs234713) that showed increased risks for spina bifida. Boyles et al [28], albeit using a different study design than ours, observed that these two SNPs were not differentially transmitted from parents of infants with spina bifida.

*MTRR *gene polymorphisms (particularly rs1801394) have been investigated as a risk factor for both spina bifida and congenital heart defects. Polymorphisms in *MTRR *could alter homocysteine levels because methionine synthase reductase participates in maintaining the vitamin B_12_-dependent conversion of homocysteine to methionine [32]. The most frequently studied *MTRR *polymorphism has been the 66A>G (rs1801394). This polymorphism in infants was associated with a 2.6-fold increased risk of spina bifida in an earlier study by us [33], it was associated with increased risk for spina bifida in another study only when vitamin B_12 _levels were low [39], or in combination with MTHFR CC genotype [35]. The polymorphism in mothers of infants with neural tube defects has been associated with increased risk in one study [40], but not in another study [41]. Recent work from the Netherlands has shown a lack of association between this polymorphism and risk for conotruncal heart defects [42] as well as no increased risks for a broader phenotypic group of heart defects [43]. In this study, the 66A>G polymorphism was not associated with increased risks for either spina bifida or conotruncal heart defects. We did observe, however, approximately 3-fold elevated risks for spina bifida associated with three other *MTRR *SNPs (rs162036, rs10380, and rs9332). The significance of these observations will have to be explored in future studies.

With respect to *MTHFD1 *and *MTHFD2*, two studies have demonstrated an association with one polymorphism (rs 2236225) in *MTHFD1 *and NTD risk. One study showed a 1.5-fold increase in risk of an NTD-affected pregnancy in Irish women who were homozygous AA [44], a finding that confirmed an earlier increased risk that was identified in Irish women. Another study showed a similar risk for Italian women as well as a 1.9-fold risk for infants with the AA genotype to have spina bifida [45]. For this particular SNP, we observed a similar magnitude of risk (OR = 1.6) for infants with the homozygous genotype, but the estimate was relatively imprecise. We did observe a modestly elevated spina bifida risk for individuals who were homozygous for another *MTHFD1 *SNP (rs2236224) and modestly lowered risks for three others (hcv11462908, rs702465, and rs7571842). These observations will need to be replicated in future studies.

Polymorphisms in the *DHFR *gene have not been well-studied for their role in risks of birth defects. Three studies have investigated a 19-bp deletion with mixed results [46-48]. That particular polymorphism was not interrogated in the current study.

Our analyses did not show associations with SNPs in *RFC1*. Previous investigations of this gene have focused on a particular SNP, rs1051266, and have found mixed results [37,41,49-53]. This particular SNP was not analyzed here as a result of too many samples failing to be genotyped for this SNP using the SNPlex platform.

Recent studies have focused on the importance of *TYMS *in the folate metabolic pathway, including associations between *TYMS *polymorphisms and folate levels [54-56]. This folate-dependent enzyme catalyzes the reductive methylation of deoxyuridylate (dUMP) to thymidylate (dTMP), thereby playing a central role in DNA synthesis and repair by serving as the primary intracellular source of dTMP [54,57-59]. We previously [56] observed a 4-fold increased risk of spina bifida in nonHispanic white infants who had a polymorphism for a 28 bp insertion in the promoter region. This observation, however, was not replicated in a population from the northern UK [55]. This particular polymorphism was not interrogated in the current study. Three of the five *TYMS *SNPs (rs284179, rs1001761, and rs502396) investigated here showed elevated risks for spina bifida for both heterozygote or homozygote individuals. This finding and the corresponding haplotype finding (Table 3) will be important to explore in future studies.

The strengths of this study were: 1) it investigated the potential effects of a large number of folate pathway SNPs, as well as investigated haplotype associations; 2) it had population-based ascertainment of two case phenotypes and controls; and 3) it included cases and controls born before the US food supply was fortified with folic acid, thus we would expect a sizable proportion of cases to have been folate-responsive.

Conversely, our study was limited in its effect estimation owing to small sample sizes for some comparisons. For example, our study had 80% power to detect risks of 2.5 or more associated with genotypes that were observed in at least 4% of controls. Another potential limitation is the lack of information on maternal folate status. Our working hypothesis is that transient elevation in maternal serum folate from supplementation or dietary intake could prevent birth defects by overcoming metabolic inefficiencies or transport-related issues. Absence of information on low folate status would make it more difficult to find putative genotypes. It is also possible that the protective effect of folic acid relates to correction of a maternal metabolic defect, rather than the fetus. Our study was limited to infant genotype information. Thus, we were unable to investigate the potential effects of maternal genotype. As with any study that seeks to explore associations with a large number of genotypes, findings are subject to chance owing to multiple comparisons. As noted above, we conducted 472 analytic comparisons and thus expected more "statistically significant" findings to arise by chance alone. Further, our findings may have been influenced by uncontrolled confounding by population stratification undetectable in analyses stratified or adjusted by race/ethnicity [60,61]. Lastly, the selected SNPs represent only a fraction of the potential variation of the studied genes. Thus, full gene coverage was not achieved even though a large number of SNPs was studied.

## Conclusion

Despite compelling evidence that folate intake by women in early pregnancy substantially reduces risks of selected birth defects, the underlying mechanisms have not been elucidated. Our study attempted to determine genetic mechanisms responsible for folic acid's preventive effects. Our observations do not implicate a particular folate transport or metabolism gene to be strongly associated with risks for spina bifida or conotruncal defects. Although we explored a sizable number of polymorphic areas in these genes, we clearly did not capture all the genetic variation. Thus, these genes may continue to be candidates for further inquiry. Alternatively, the preventive role of folate may be via other biological mechanisms such as methylation of nonfolate-related genes that participate in the closure of the neural tube or the development of the heart.

## Competing interests

The authors declare that they have no competing interests.

## Authors' contributions

GMS conceived of the study and participated in the statistical analysis. WL conducted the molecular genetic studies. HZ conducted the molecular genetic studies and participated in the statistical analysis. WY conducted the statistical analysis. FBSB conducted the statistical analysis. SLC participated in the statistical analysis. LFB designed and participated in the statistical analysis. EJL conceived of the study and participated in the statistical analysis. RHF conceived of the study and directed the laboratory molecular genetic studies. All authors read and approved the final manuscript.

## Pre-publication history

The pre-publication history for this paper can be accessed here:



## Supplementary Material

Additional file 1 **Appendix**. Risks of spina bifida and conotruncal heart defects among California infants associated with 118 SNPs in 14 genes involved in folate metabolism or transport relative to nonmalformed population-based controls. Click here for file
