# Supplementary material for: 118 SNPs of folate-related genes and risks of spina bifida and conotruncal heart defects
Source: BMC Med Genet. 2009 Jun 3;10:49. doi: 10.1186/1471-2350-10-49 (PMC2700092; doi:10.1186/1471-2350-10-49)
Supplement: Additional file 1 — Appendix. Risks of spina bifida and conotruncal heart defects among California infants associated with 118 SNPs in 14 genes involved in folate metabolism or transport relative to nonmalformed population-based controls. [file 1471-2350-10-49-S1.doc]

Appendix 1. Risks of spina bifida and conotruncal heart defects among California infants associated with 118 SNPs in 14 genes involved in folate metabolism or transport relative to nonmalformed population-based controls.

| **Gene** | **Genotype** | **SNP ID RS#** | **Control Freq (%)^1^** | **Spina bifida Odds Ratio** | **95% CI** | **ConotruncalOdds Ratio** | **95% CI** |
| --- | --- | --- | --- | --- | --- | --- | --- |
| *BHMT* | Heterozygote | rs3733890 | 44.0 | 1.3 | 0.9-1.9 | 1.1 | 0.7-1.6 |
|  | Homozygote |  | 9.5 | **1.8** | 1.1-3.1 | 0.9 | 0.5-1.7 |
|  | Reference |  | 46.5 | Ref |  | Ref |  |
| *BHMT* | Heterozygote | rs1915706 | 49.6 | 0.8 | 0.5-1.2 | 0.7 | 0.4-1.1 |
|  | Homozygote |  | 22.6 | 1.3 | 0.8-2.1 | 0.8 | 0.5-1.3 |
|  | Reference |  | 24.2 | Ref |  | Ref |  |
| *BHMT* | Heterozygote | rs1316753 | 50.1 | 0.8 | 0.5-1.1 | 1.0 | 0.7-1.6 |
|  | Homozygote |  | 20.9 | 0.8 | 0.5-1.3 | 1.2 | 0.7-2.1 |
|  | Reference |  | 29.0 | Ref |  | Ref |  |
| *BHMT* | Heterozygote | rs617219 | 48.8 | 0.7 | 0.5-1.0 | 1.1 | 0.7-1.7 |
|  | Homozygote |  | 13.7 | 0.8 | 0.5-1.4 | 1.1 | 0.6-2.1 |
|  | Reference |  | 34.0 | Ref |  | Ref |  |
| *BHMT* | Heterozygote | rs645112 | 51.0 | 1.0 | 0.7-1.4 | 0.9 | 0.6-1.4 |
|  | Homozygote |  | 15.0 | 1.2 | 0.7-1.9 | 1.4 | 0.8-2.5 |
|  | Reference |  | 30.9 | Ref |  | Ref |  |
| *BHMT* | Heterozygote | rs585800 | 29.8 | 0.7 | 0.5-1.0 | 0.7 | 0.5-1.1 |
|  | Homozygote |  | 2.2 | 1.2 | 0.5-3.4 | 1.1 | 0.4-3.4 |
|  | Reference |  | 62.1 | Ref |  | Ref |  |
| *BHMT* | Heterozygote | rs3829809 | 49.3 | 0.7 | 0.5-1.0 | 1.0 | 0.6-1.4 |
|  | Homozygote |  | 12.0 | 0.9 | 0.5-1.5 | 1.2 | 0.6-2.3 |
|  | Reference |  | 38.7 | Ref |  | Ref |  |
| *BHMT* | Heterozygote | rs567754 | 43.5 | 0.7 | 0.5-1.0 | 1.0 | 0.6-1.5 |
|  | Homozygote |  | 12.0 | 0.7 | 0.4-1.3 | 1.2 | 0.6-2.3 |
|  | Reference |  | 40.4 | Ref |  | Ref |  |
| *BHMT2* | Heterozygote | rs642431 | 30.6 | 1.0 | 0.7-1.5 | 1.0 | 0.6-1.5 |
|  | Homozygote |  | 6.1 | 1.0 | 0. 5-2.0 | 1.5 | 0.7-3.5 |
|  | Reference |  | 54.3 | Ref |  | Ref |  |
| *BHMT2* | Heterozygote | rs626105 | 37.3 | 0.9 | 0.6-1.2 | 0.9 | 0.6-1.3 |
|  | Homozygote |  | 3.9 | 1.1 | 0.5-2.5 | 2.2 | 0.8-5.9 |
|  | Reference |  | 54.9 | Ref |  | Ref |  |
| *BHMT2* | Heterozygote | rs682985 | 47.9 | 1.0 | 0.7-1.5 | 0.9 | 0.6-1.5 |
|  | Homozygote |  | 14.5 | 1.1 | 0.7-1.8 | 1.4 | 0.8-2.4 |
|  | Reference |  | 33.2 | Ref |  | Ref |  |
| *BHMT2* | Heterozygote | rs2253262 | 44.6 | 1.1 | 0.8-1.5 | 0.9 | 0.6-1.4 |
|  | Homozygote |  | 10.3 | 1.1 | 0.7-2.0 | 1.5 | 0.8-2.9 |
|  | Reference |  | 41.5 | Ref |  | Ref |  |
| *BHMT2* | Heterozygote | rs670220 | 36.8 | 0.9 | 0.7-1.3 | 0.9 | 0.6-1.3 |
|  | Homozygote |  | 3.9 | 0.9 | 0. 4-2.2 | 2.4 | 0.9-6.3 |
|  | Reference |  | 56.0 | Ref |  | Ref |  |
| *BHMT2* | Heterozygote | rs592052 | 48.8 | 0.9 | 0.6-1.4 | 0.9 | 0.6-1.4 |
|  | Homozygote |  | 25.1 | 0.9 | 0.6-1.4 | 1.1 | 0.6-1.9 |
|  | Reference |  | 25.4 | Ref |  | Ref |  |
| *BHMT2* | Heterozygote | rs597560 | 50.7 | 1.0 | 0.7-1.4 | 0.9 | 0.6-1.4 |
|  | Homozygote |  | 15.0 | 1.1 | 0.7-1.8 | 1.4 | 0.8-2.5 |
|  | Reference |  | 32.6 | Ref |  | Ref |  |
| *CBS* | Heterozygote | rs2851391 | 40.4 | **1.8** | 1.2-2.7 | 1.0 | 0.7-1.6 |
|  | Homozygote |  | 22.0 | **2.0** | 1.2-3.1 | 0.8 | 0.5-1.5 |
|  | Reference |  | 30.1 | Ref |  | Ref |  |
| *CBS* | Heterozygote | rs2298759 | 0.8 | 2.1 | 0.5-9.4 | 1.6 | 0.1-25.5 |
|  | Homozygote |  | 0.3 | 1.6 | 0.1-25.1 | - |  |
|  | Reference |  | 71.3 | Ref |  | Ref |  |
| *CBS* | Heterozygote | rs234714 | 32.9 | 1.2 | 0.9-1.8 | 1.1 | 0.7-1.7 |
|  | Homozygote |  | 10.6 | 1.7 | 1.0-2.8 | 0.9 | 0.4-2.1 |
|  | Reference |  | 46.5 | Ref |  | Ref |  |
| *CBS* | Heterozygote | rs1051319 | 17.8 | 0.8 | 0.5-1.2 | 1.1 | 0.7-1.9 |
|  | Homozygote |  | 1.7 | 0.8 | 0.2-3.0 | 2.6 | 0.9-7.6 |
|  | Reference |  | 72.4 | Ref |  | Ref |  |
| *CBS* | Heterozygote | rs234784 | 47.1 | 1.1 | 0.7-1.5 | 0.8 | 0.5-1.2 |
|  | Homozygote |  | 12.5 | 1.2 | 0.7-2.0 | 1.1 | 0.6-1.9 |
|  | Reference |  | 40.1 | Ref |  | Ref |  |
| *CBS* | Heterozygote | rs12613 | 9.8 | 1.1 | 0.7-1.9 | 1.6 | 0.9-2.9 |
|  | Homozygote |  | 0.0 | - |  | - |  |
|  | Reference |  | 82.7 | Ref |  | Ref |  |
| *CBS* | Heterozygote | rs234785 | 43.7 | 0.7 | 0.5-1.0 | 0.8 | 0.6-1.3 |
|  | Homozygote |  | 13.7 | 0.6 | 0.3-1.0 | 0.8 | 0.4-1.4 |
|  | Reference |  | 42.6 | Ref |  | Ref |  |
| *CBS* | Heterozygote | rs234713 | 34.3 | 0.9 | 0.6-1.3 | 0.7 | 0.5-1.1 |
|  | Homozygote |  | 2.5 | **2.9** | 1.3-6.7 | 2.3 | 0.8-7.0 |
|  | Reference |  | 54.3 | Ref |  | Ref |  |
| *CBS* | Heterozygote | rs234783 | 46.2 | 0.9 | 0.6-1.3 | 1.0 | 0.6-1.5 |
|  | Homozygote |  | 21.7 | 0.8 | 0.5-1.2 | 0.8 | 0.5-1.4 |
|  | Reference |  | 32.0 | Ref |  | Ref |  |
| *DHFR* | Heterozygote | rs1650697 | 12.5 | 0.8 | 0.5-1.3 | 1.0 | 0.6-1.8 |
|  | Homozygote |  | 4.5 | 1.7 | 0.9-3.4 | 1.5 | 0.7-3.4 |
|  | Reference |  | 75.2 | Ref |  | Ref |  |
| *DHFR* | Heterozygote | rs12109877 | 2.5 | 1.2 | 0.5-3.2 | 0.6 | 0.2-2.0 |
|  | Homozygote |  | 1.4 | 1.4 | 0.4-4.7 | - |  |
|  | Reference |  | 90.3 | Ref |  | Ref |  |
| *DHFR* | Heterozygote | rs380691 | 46.0 | 0.7 | 0.5-1.0 | 0.7 | 0.5-1.1 |
|  | Homozygote |  | 15.0 | 1.0 | 0.6-1.6 | 1.3 | 0.7-2.3 |
|  | Reference |  | 34.5 | Ref |  | Ref |  |
| *DHFR* | Heterozygote | rs1478834 | 27.6 | 1.1 | 0.7-1.5 | 1.1 | 0.7-1.6 |
|  | Homozygote |  | 5.3 | 0.9 | 0.5-2.0 | 1.4 | 0.6-3.2 |
|  | Reference |  | 63.5 | Ref |  | Ref |  |
| *DHFR* | Heterozygote | rs1643638 | 26.5 | 1.0 | 0.7-1.5 | 0.9 | 0.6-1.5 |
|  | Homozygote |  | 5.9 | 0.7 | 0.4-1.6 | 1.2 | 0.5-2.6 |
|  | Reference |  | 60.5 | Ref |  | Ref |  |
| *DHFR* | Heterozygote | rs2618372 | 29.3 | 1.0 | 0.7-1.4 | 1.0 | 0.6-1.5 |
|  | Homozygote |  | 5.6 | 0.8 | 0.4-1.7 | 1.2 | 0.5-2.7 |
|  | Reference |  | 62.1 | Ref |  | Ref |  |
| *DHFR* | Heterozygote | rs13161245 | 28.1 | 1.0 | 0.7-1.5 | 1.0 | 0.7-1.5 |
|  | Homozygote |  | 5.6 | 0.7 | 0.3-1.6 | 1.2 | 0.5-2.7 |
|  | Reference |  | 62.4 | Ref |  | Ref |  |
| *DHFR* | Heterozygote | rs1643650 | 27.6 | 1.0 | 0.7-1.5 | 1.1 | 0.7-1.7 |
|  | Homozygote |  | 5.6 | 0.8 | 0.4-1.8 | 1.2 | 0.6-2.7 |
|  | Reference |  | 61.6 | Ref |  | Ref |  |
| *DHFR* | Heterozygote | rs836821 | 29.3 | 1.0 | 0.7-1.4 | 1.0 | 0.7-1.5 |
|  | Homozygote |  | 5.6 | 0.9 | 0.4-1.8 | 1.3 | 0.6-2.8 |
|  | Reference |  | 62.7 | Ref |  | Ref |  |
| *FOLR1* | Heterozygote | rs1540087 | 6.1 | 0.8 | 0.4-1.6 | 1.2 | 0.6-2.5 |
|  | Homozygote |  | 0.6 | 0.7 | 0.1-7.3 | 1.0 | 0.1-16.5 |
|  | Reference |  | 89.1 | Ref |  | Ref |  |
| *FOLR1* | Heterozygote | rs11235462 | 27.9 | 1.3 | 0.9-1.9 | 1.4 | 0.9-2.1 |
|  | Homozygote |  | 5.3 | 1.3 | 0.6-2.6 | 1.1 | 0.5-2.4 |
|  | Reference |  | 66.9 | Ref |  | Ref |  |
| *FOLR1* | Heterozygote | rs2071010 | 13.4 | 1.0 | 0.7-1.6 | 0.7 | 0.4-1.3 |
|  | Homozygote |  | 1.4 | 0.5 | 0.1-2.7 | 0.6 | 0.1-3.8 |
|  | Reference |  | 77.2 | Ref |  | Ref |  |
| *FOLR2* | Heterozygote | rs2298444 | 34.0 | 1.0 | 0.7-1.4 | 1.0 | 0.7-1.5 |
|  | Homozygote |  | 5.9 | 1.0 | 0.5-2.0 | 1.0 | 0.5-2.2 |
|  | Reference |  | 52.4 | Ref |  | Ref |  |
| *FOLR2* | Heterozygote | rs514933 | 44.9 | 0.9 | 0.6-1.3 | 0.8 | 0.5-1.2 |
|  | Homozygote |  | 13.4 | 1.1 | 0.7-1.8 | 1.3 | 0.7-2.2 |
|  | Reference |  | 41.8 | Ref |  | Ref |  |
| *FOLR2* | Heterozygote | rs651646 | 48.8 | 0.9 | 0.6-1.3 | 0.8 | 0.5-1.2 |
|  | Homozygote |  | 20.3 | 1.1 | 0.7-1.7 | 1.0 | 0.6-1.7 |
|  | Reference |  | 30.9 | Ref |  | Ref |  |
| *MTHFD1* | Heterozygote | rs2236222 | 16.2 | 1.1 | 0.7-1.7 | 1.1 | 0.6-1.8 |
|  | Homozygote |  | 0.8 | - |  | 5.3 | 0.6-45.6 |
|  | Reference |  | 78.6 | Ref |  | Ref |  |
| *MTHFD1* | Heterozygote | rs2236224 | 47.4 | 1.3 | 0.9-1.9 | 0.8 | 0.5-1.3 |
|  | Homozygote |  | 15.9 | **1.7** | 1.1-2.7 | 1.2 | 0.7-2.0 |
|  | Reference |  | 34.5 | Ref |  | Ref |  |
| *MTHFD1* | Heterozygote | rs1950902 | 26.5 | 1.0 | 0.7-1.5 | 0.8 | 0.5-1.3 |
|  | Homozygote |  | 4.7 | 0.4 | 0.1-1.0 | 1.0 | 0.4-2.4 |
|  | Reference |  | 59.3 | Ref |  | Ref |  |
| *MTHFD1* | Heterozygote | rs2236225 | 49.9 | 1.1 | 0.8-1.7 | 0.8 | 0.6-1.3 |
|  | Homozygote |  | 18.7 | 1.6 | 1.0-2.5 | 0.9 | 0.5-1.5 |
|  | Reference |  | 31.5 | Ref |  | Ref |  |
| *MTHFD1* | Heterozygote | hCV11462908 | 28.1 | 1.1 | 0.7-1.5 | 1.1 | 0.7-1.7 |
|  | Homozygote |  | 3.9 | **0.2** | 0.0-0.9 | 0.7 | 0.2-2.4 |
|  | Reference |  | 68.0 | Ref |  | Ref |  |
| *MTHFD1* | Heterozygote | hCV11660794 | 22.6 | 1.3 | 0.9-1.9 | 0.8 | 0.5-1.2 |
|  | Homozygote |  | 5.3 | 0.5 | 0.2-1.2 | 1.0 | 0.3-3.0 |
|  | Reference |  | 67.4 | Ref |  | Ref |  |
| *MTHFD1* | Heterozygote | rs11849530 | 31.5 | 1.2 | 0.8-1.7 | 1.1 | 0.7-1.6 |
|  | Homozygote |  | 7.0 | 0.6 | 0.3-1.3 | 0.8 | 0.4-1.8 |
|  | Reference |  | 57.4 | Ref |  | Ref |  |
| *MTHFD1* | Heterozygote | rs1256146 | 26.2 | 1.1 | 0.7-1.5 | 1.0 | 0.7-1.6 |
|  | Homozygote |  | 3.6 | 0.3 | 0.1-1.1 | 0.4 | 0.1-2.1 |
|  | Reference |  | 65.2 | Ref |  | Ref |  |
| *MTHFD1* | Heterozygote | rs10137921 | 1.4 | 1.6 | 0.5-5.4 | 2.1 | 0.5-8.5 |
|  | Homozygote |  | 0.0 | - |  | - |  |
|  | Reference |  | 95.0 | Ref |  | Ref |  |
| *MTHFD1* | Heterozygote | rs1256142 | 49.0 | 1.2 | 0.8-1.8 | 1.0 | 0.6-1.5 |
|  | Homozygote |  | 23.4 | 1.5 | 0.9-2.4 | 0.8 | 0.5-1.4 |
|  | Reference |  | 25.4 | Ref |  | Ref |  |
| *MTHFD2* | Heterozygote | rs11126426 | 48.2 | 0.8 | 0.6-1.1 | 0.9 | 0.6-1.4 |
|  | Homozygote |  | 16.2 | 1.3 | 0.8-2.0 | 0.9 | 0.5-1.6 |
|  | Reference |  | 35.7 | Ref |  | Ref |  |
| *MTHFD2* | Heterozygote | rs702465 | 47.6 | **0.6** | 0.4-0.9 | 0.8 | 0.5-1.2 |
|  | Homozygote |  | 15.6 | 0.8 | 0.5-1.4 | 1.1 | 0.6-1.9 |
|  | Reference |  | 33.4 | Ref |  | Ref |  |
| *MTHFD2* | Heterozygote | rs1667599 | 16.2 | 1.1 | 0.7-1.7 | 0.7 | 0.4-1.2 |
|  | Homozygote |  | 0.8 | 0.5 | 0.0-4.5 | 2.0 | 0.5-8.0 |
|  | Reference |  | 83.0 | Ref |  | Ref |  |
| *MTHFD2* | Heterozygote | rs1667627 | 47.4 | 0.8 | 0.5-1.1 | 0.9 | 0.6-1.4 |
|  | Homozygote |  | 15.0 | 0.9 | 0.6-1.4 | 1.3 | 0.7-2.2 |
|  | Reference |  | 33.7 | Ref |  | Ref |  |
| *MTHFD2* | Heterozygote | rs828858 | 41.8 | 0.8 | 0.6-1.1 | 0.9 | 0.6-1.3 |
|  | Homozygote |  | 7.5 | 1.2 | 0.7-2.2 | 1.1 | 0.5-2.2 |
|  | Reference |  | 50.7 | Ref |  | Ref |  |
| *MTHFD2* | Heterozygote | rs702466 | 42.9 | 0.9 | 0.7-1.3 | 1.0 | 0.7-1.5 |
|  | Homozygote |  | 8.6 | 1.5 | 0.9-2.6 | 1.1 | 0.6-2.2 |
|  | Reference |  | 48.2 | Ref |  | Ref |  |
| *MTHFD2* | Heterozygote | rs7571842 | 47.6 | **0.6** | 0.4-0.9 | 0.9 | 0.6-1.5 |
|  | Homozygote |  | 21.7 | 0.8 | 0.5-1.2 | 1.4 | 0.8-2.3 |
|  | Reference |  | 30.6 | Ref |  | Ref |  |
| *MTHFD2* | Heterozygote | rs828903 | 45.1 | 0.8 | 0.6-1.2 | 1.0 | 0.6-1.5 |
|  | Homozygote |  | 11.7 | 1.4 | 0.9-2.4 | 1.4 | 0.7-2.6 |
|  | Reference |  | 37.6 | Ref |  | Ref |  |
| *MTHFR* | Heterozygote | rs3737964 | 30.1 | 1.0 | 0.7-1.5 | 1.1 | 0.8-1.7 |
|  | Homozygote |  | 3.6 | 0.7 | 0.3-1.9 | 1.2 | 0.5-3.0 |
|  | Reference |  | 62.1 | Ref |  | Ref |  |
| *MTHFR* | Heterozygote | rs535107 | 40.7 | 0.8 | 0.6-1.1 | 1.0 | 0.7-1.6 |
|  | Homozygote |  | 14.2 | 0.7 | 0.4-1.2 | 0.8 | 0.4-1.4 |
|  | Reference |  | 38.4 | Ref |  | Ref |  |
| *MTHFR* | Heterozygote | rs1931226 | 0.0 | - |  | - |  |
|  | Homozygote |  | 0.0 | - |  | - |  |
|  | Reference |  | 97.0 | Ref |  | Ref |  |
| *MTHFR* | Heterozygote | rs4846048 | 29.0 | 1.0 | 0.7-1.4 | 1.1 | 0.7-1.6 |
|  | Homozygote |  | 9.2 | 0.9 | 0.5-1.6 | 1.4 | 0.7-2.7 |
|  | Reference |  | 51.5 | Ref |  | Ref |  |
| *MTHFR* | Heterozygote | rs7525338 | 0.0 | - |  | - |  |
|  | Homozygote |  | 0.0 | - |  | - |  |
|  | Reference |  | 97.5 | Ref |  | Ref |  |
| *MTHFR* | Heterozygote | rs2274976 | 12.3 | 0.6 | 0.3-1.0 | 0.8 | 0.4-1.4 |
|  | Homozygote |  | 0.8 | - |  | - |  |
|  | Reference |  | 79.9 | Ref |  | Ref |  |
| *MTHFR* | Heterozygote | rs4846052 | 42.1 | 0.8 | 0.6-1.1 | 1.0 | 0.7-1.6 |
|  | Homozygote |  | 17.8 | 0.8 | 0.5-1.3 | 0.9 | 0.5-1.5 |
|  | Reference |  | 37.1 | Ref |  | Ref |  |
| *MTHFR* | Heterozygote | rs1801133 | 42.9 | 1.2 | 0.8-1.7 | 0.9 | 0.6-1.4 |
|  | Homozygote |  | 12.5 | **2.0** | 1.2-3.1 | 0.9 | 0.5-1.8 |
|  | Reference |  | 44.0 | Ref |  | Ref |  |
| *MTHFR* | Heterozygote | rs1889292 | 44.3 | 0.8 | 0.5-1.1 | 1.1 | 0.7-1.6 |
|  | Homozygote |  | 13.9 | 0.7 | 0.4-1.2 | 0.9 | 0.5-1.7 |
|  | Reference |  | 41.8 | Ref |  | Ref |  |
| *MTHFR* | Heterozygote | rs2066470 | 18.7 | 0.7 | 0.4-1.0 | 0.7 | 0.4-1.2 |
|  | Homozygote |  | 1.4 | 0.7 | 0.2-3.1 | 0.3 | 0.0-3.0 |
|  | Reference |  | 75.2 | Ref |  | Ref |  |
| *MTHFR* | Heterozygote | rs4846051 | 3.6 | 1.2 | 0.6-2.7 | 1.4 | 0.6-3.3 |
|  | Homozygote |  | 0.3 | - |  | - |  |
|  | Reference |  | 89.1 | Ref |  | Ref |  |
| *MTHFR* | Heterozygote | rs1476413 | 35.9 | 0.7 | 0.5-1.0 | 0.7 | 0.4-1.0 |
|  | Homozygote |  | 5.0 | 0.6 | 0.3-1.4 | 1.2 | 0.5-3.1 |
|  | Reference |  | 52.9 | Ref |  | Ref |  |
| *MTHFR* | Heterozygote | rs1801131 | 36.2 | 0.8 | 0.6-1.1 | 0.9 | 0.6-1.3 |
|  | Homozygote |  | 9.8 | 0.7 | 0.4-1.3 | 0.6 | 0.3-1.3 |
|  | Reference |  | 53.8 | Ref |  | Ref |  |
| *MTR* | Heterozygote | rs2275565 | 34.8 | 1.0 | 0.7-1.5 | 1.1 | 0.7-1.6 |
|  | Homozygote |  | 6.1 | 0.7 | 0.4-1.5 | 1.5 | 0.7-3.0 |
|  | Reference |  | 55.2 | Ref |  | Ref |  |
| *MTR* | Heterozygote | rs1806505 | 45.4 | 1.4 | 1.0-1.9 | 1.0 | 0.7-1.6 |
|  | Homozygote |  | 13.9 | 1.3 | 0.8-2.2 | 1.0 | 0.6-1.7 |
|  | Reference |  | 38.2 | Ref |  | Ref |  |
| *MTR* | Heterozygote | rs3820571 | 44.3 | 1.0 | 0.7-1.5 | 0.9 | 0.6-1.3 |
|  | Homozygote |  | 8.4 | 1.2 | 0.7-2.2 | 1.0 | 0.5-2.0 |
|  | Reference |  | 43.5 | Ref |  | Ref |  |
| *MTR* | Heterozygote | rs3754255 | 46.5 | 1.2 | 0.8-1.8 | 1.1 | 0.7-1.7 |
|  | Homozygote |  | 18.1 | 1.2 | 0.8-2.0 | 1.1 | 0.6-1.8 |
|  | Reference |  | 30.1 | Ref |  | Ref |  |
| *MTR* | Heterozygote | rs10802569 | 46.0 | 0.9 | 0.7-1.3 | 0.8 | 0.5-1.2 |
|  | Homozygote |  | 12.8 | 0.8 | 0.5-1.3 | 0.9 | 0.5-1.7 |
|  | Reference |  | 37.3 | Ref |  | Ref |  |
| *MTR* | Heterozygote | rs1266164 | 43.7 | 1.0 | 0.7-1.4 | 0.8 | 0.5-1.2 |
|  | Homozygote |  | 8.4 | 1.1 | 0.6-2.0 | 0.9 | 0.5-1.8 |
|  | Reference |  | 44.0 | Ref |  | Ref |  |
| *MTR* | Heterozygote | rs1805087 | 31.2 | 1.0 | 0.7-1.4 | 1.0 | 0.7-1.5 |
|  | Homozygote |  | 3.3 | 0.9 | 0.4-2.2 | 1.6 | 0.6-4.4 |
|  | Reference |  | 61.8 | Ref |  | Ref |  |
| *MTR* | Heterozygote | rs4659743 | 46.2 | 0.9 | 0.7-1.3 | 0.8 | 0.5-1.2 |
|  | Homozygote |  | 7.8 | 1.3 | 0.7-2.2 | 0.8 | 0.4-1.7 |
|  | Reference |  | 44.3 | Ref |  | Ref |  |
| *MTR* | Heterozygote | rs6676866 | 50.0 | 0.9 | 0.6-1.3 | 0.8 | 0.5-1.2 |
|  | Homozygote |  | 17.0 | 0.7 | 0.5-1.2 | 1.0 | 0.5-1.7 |
|  | Reference |  | 31.8 | Ref |  | Ref |  |
| *MTR* | Heterozygote | rs12060570 | 46.2 | 1.3 | 0.9-1.8 | 1.0 | 0.7-1.5 |
|  | Homozygote |  | 14.5 | 1.2 | 0.7-2.0 | 0.9 | 0.5-1.6 |
|  | Reference |  | 37.9 | Ref |  | Ref |  |
| *MTR* | Heterozygote | rs955516 | 45.7 | 1.3 | 0.9-1.9 | 1.0 | 0.6-1.5 |
|  | Homozygote |  | 15.3 | 1.1 | 0.7-1.8 | 0.8 | 0.5-1.4 |
|  | Reference |  | 38.2 | Ref |  | Ref |  |
| *MTR* | Heterozygote | rs4077829 | 44.9 | 1.3 | 0.9-1.9 | 0.9 | 0.6-1.4 |
|  | Homozygote |  | 14.8 | 1.1 | 0.7-1.8 | 0.8 | 0.5-1.4 |
|  | Reference |  | 37.1 | Ref |  | Ref |  |
| *MTR* | Heterozygote | rs1770449 | 43.2 | 0.9 | 0.6-1.3 | 0.8 | 0.5-1.2 |
|  | Homozygote |  | 8.1 | 1.1 | 0.6-1.9 | 0.8 | 0.4-1.7 |
|  | Reference |  | 43.2 | Ref |  | Ref |  |
| *MTR* | Heterozygote | rs3768139 | 44.9 | 0.9 | 0.7-1.3 | 0.8 | 0.5-1.1 |
|  | Homozygote |  | 7.5 | 1.3 | 0.7-2.3 | 0.9 | 0.5-1.9 |
|  | Reference |  | 43.2 | Ref |  | Ref |  |
| *MTR* | Heterozygote | rs4659724 | 44.6 | 1.2 | 0.9-1.8 | 0.9 | 0.6-1.4 |
|  | Homozygote |  | 14.2 | 1.1 | 0.7-1.9 | 0.8 | 0.4-1.4 |
|  | Reference |  | 38.4 | Ref |  | Ref |  |
| *MTR* | Heterozygote | rs6668344 | 45.4 | 1.2 | 0.9-1.8 | 1.0 | 0.6-1.5 |
|  | Homozygote |  | 14.5 | 1.2 | 0.7-1.9 | 0.9 | 0.5-1.6 |
|  | Reference |  | 36.5 | Ref |  | Ref |  |
| *MTR* | Heterozygote | rs7367859 | 40.7 | 1.0 | 0.7-1.5 | 0.8 | 0.5-1.3 |
|  | Homozygote |  | 10.9 | 0.5 | 0.3-1.0 | 0.9 | 0.5-1.7 |
|  | Reference |  | 42.3 | Ref |  | Ref |  |
| *MTR* | Heterozygote | rs3768142 | 45.1 | 1.0 | 0.7-1.4 | 0.8 | 0.5-1.2 |
|  | Homozygote |  | 12.8 | 0.8 | 0.5-1.4 | 1.0 | 0.6-1.9 |
|  | Reference |  | 38.2 | Ref |  | Ref |  |
| *MTR* | Heterozygote | rs10925252 | 44.6 | 1.3 | 0.9-1.8 | 1.0 | 0.6-1.5 |
|  | Homozygote |  | 15.0 | 1.1 | 0.7-1.8 | 0.9 | 0.5-1.5 |
|  | Reference |  | 37.3 | Ref |  | Ref |  |
| *MTR* | Heterozygote | rs2229276 | 44.3 | 1.4 | 1.0-2.0 | 1.1 | 0.7-1.6 |
|  | Homozygote |  | 14.5 | 1.3 | 0.8-2.1 | 0.8 | 0.5-1.5 |
|  | Reference |  | 36.2 | Ref |  | Ref |  |
| *MTR* | Heterozygote | rs1050993 | 44.6 | 0.9 | 0.7-1.3 | 0.9 | 0.6-1.3 |
|  | Homozygote |  | 8.4 | 1.2 | 0.7-2.1 | 0.9 | 0.4-1.7 |
|  | Reference |  | 44.3 | Ref |  | Ref |  |
| *MTRR* | Heterozygote | rs162036 | 30.4 | 1.1 | 0.8-1.6 | 1.0 | 0.7-1.5 |
|  | Homozygote |  | 3.9 | **3.0** | 1.5-5.9 | 1.4 | 0.4-4.6 |
|  | Reference |  | 61.3 | Ref |  | Ref |  |
| *MTRR* | Heterozygote | rs16879334 | 12.5 | 1.2 | 0.7-1.9 | 1.2 | 0.6-2.2 |
|  | Homozygote |  | 0.3 | 4.4 | 0. 5-42.3 | - |  |
|  | Reference |  | 77.2 | Ref |  | Ref |  |
| *MTRR* | Heterozygote | rs1802059 | 38.7 | 0.9 | 0.6-1.2 | 0.9 | 0.6-1.4 |
|  | Homozygote |  | 6.7 | 1.1 | 0.6-2.1 | 1.0 | 0.5-2.1 |
|  | Reference |  | 49.3 | Ref |  | Ref |  |
| *MTRR* | Heterozygote | rs2287779 | 14.2 | 1.0 | 0.6-1.5 | 1.3 | 0.7-2.5 |
|  | Homozygote |  | 0.0 | - |  | - |  |
|  | Reference |  | 83.0 | Ref |  | Ref |  |
| *MTRR* | Heterozygote | rs326120 | 24.5 | 0.9 | 0.6-1.4 | 1.1 | 0.7-1.7 |
|  | Homozygote |  | 2.5 | 1.1 | 0.4-3.0 | 0.6 | 0.1-2.2 |
|  | Reference |  | 60.7 | Ref |  | Ref |  |
| *MTRR* | Heterozygote | rs10380 | 30.1 | 1.1 | 0.8-1.6 | 0.9 | 0.6-1.3 |
|  | Homozygote |  | 3.1 | **3.4** | 1.6-7.1 | 2.9 | 0.6-14.8 |
|  | Reference |  | 63.2 | Ref |  | Ref |  |
| *MTRR* | Heterozygote | rs1801394 | 44.9 | **0.7** | 0.5-0.9 | 1.0 | 0.7-1.6 |
|  | Homozygote |  | 18.1 | 0.7 | 0.4-1.1 | 0.8 | 0.4-1.3 |
|  | Reference |  | 33.7 | Ref |  | Ref |  |
| *MTRR* | Heterozygote | rs9332 | 29.3 | 1.2 | 0.8-1.7 | 1.0 | 0.6-1.5 |
|  | Homozygote |  | 3.9 | **2.7** | 1.3-5.3 | 1.4 | 0.4-4.5 |
|  | Reference |  | 59.1 | Ref |  | Ref |  |
| *MTRR* | Heterozygote | rs10064631 | 1.4 | 3.0 | 1.0-8.7 | 4.8 | 1.0-22.3 |
|  | Homozygote |  | 0.6 | - |  | - |  |
|  | Reference |  | 93.0 | Ref |  | Ref |  |
| *MTRR* | Heterozygote | rs2303080 | 10.0 | 0.9 | 0.5-1.6 | 1.1 | 0.5-2.4 |
|  | Homozygote |  | 0.3 | 4.0 | 0.4-39.0 | 2.1 | 0.2-23.1 |
|  | Reference |  | 85.8 | Ref |  | Ref |  |
| *MTRR* | Heterozygote | rs3776455 | 46.2 | 1.0 | 0.7-1.5 | 0.8 | 0.5-1.3 |
|  | Homozygote |  | 23.4 | 1.6 | 1.0-2.5 | 1.0 | 0.6-1.8 |
|  | Reference |  | 25.4 | Ref |  | Ref |  |
| *MTRR* | Heterozygote | rs1532268 | 39.3 | 0.8 | 0.6-1.2 | 0.9 | 0.6-1.3 |
|  | Homozygote |  | 6.4 | 1.2 | 0.6-2.3 | 1.0 | 0.5-2.1 |
|  | Reference |  | 49.3 | Ref |  | Ref |  |
| *MTRR* | Heterozygote | rs162048 | 27.9 | 1.0 | 0.7-1.5 | 1.2 | 0.8-1.8 |
|  | Homozygote |  | 3.9 | 0.6 | 0.2-1.6 | 0.4 | 0.2-1.3 |
|  | Reference |  | 66.9 | Ref |  | Ref |  |
| *NOS3* | Heterozygote | rs891512 | 24.5 | 0.8 | 0.5-1.2 | 0.7 | 0.4-1.1 |
|  | Homozygote |  | 2.5 | 0.7 | 0.2-2.0 | 1.5 | 0.5-4.3 |
|  | Reference |  | 59.6 | Ref |  | Ref |  |
| *NOS3* | Heterozygote | rs1800779 | 37.1 | 0.9 | 0.6-1.3 | 0.8 | 0.6-1.3 |
|  | Homozygote |  | 5.6 | 1.0 | 0.5-2.1 | 1.0 | 0.4-2.0 |
|  | Reference |  | 44.9 | Ref |  | Ref |  |
| *NOS3* | Heterozygote | rs3918211 | 3.3 | 0.2 | 0.1-1.0 | 0.3 | 0.1-1.7 |
|  | Homozygote |  | 0.0 | - |  |  |  |
|  | Reference |  | 93.6 | Ref |  | Ref |  |
| *RFC1* | Heterozygote | rs3788189 | 37.6 | 1.2 | 0.8-1.9 | 1.1 | 0.6-2.0 |
|  | Homozygote |  | 20.1 | 1.3 | 0.8-2.2 | 1.6 | 0.9-3.0 |
|  | Reference |  | 23.4 | Ref |  | Ref |  |
| *RFC1* | Heterozygote | rs12483377 | 11.4 | 1.1 | 0.7-1.8 | 1.6 | 0.9-2.8 |
|  | Homozygote |  | 0.8 | - |  | 0.4 | 0.0-3.5 |
|  | Reference |  | 87.7 | Ref |  | Ref |  |
| *RFC1* | Heterozygote | rs2236484 | 47.6 | 0.9 | 0.6-1.4 | 0.7 | 0.5-1.1 |
|  | Homozygote |  | 20.6 | 1.1 | 0.7-1.7 | 1.0 | 0.6-1.7 |
|  | Reference |  | 30.4 | Ref |  | Ref |  |
| *RFC1* | Heterozygote | rs3788190 | 42.6 | 1.1 | 0.7-1.6 | 1.0 | 0.6-1.5 |
|  | Homozygote |  | 20.9 | 1.0 | 0.6-1.6 | 1.0 | 0.6-1.8 |
|  | Reference |  | 27.6 | Ref |  | Ref |  |
| *RFC1* | Heterozygote | rs10483080 | 23.1 | 1.3 | 0.9-1.8 | 1.1 | 0.7-1.7 |
|  | Homozygote |  | 2.2 | 0.2 | 0.0-1.5 | 0.5 | 0.1-2.1 |
|  | Reference |  | 74.4 | Ref |  | Ref |  |
| *RFC1* | Heterozygote | rs2330183 | 42.3 | 1.1 | 0.7-1.6 | 0.8 | 0.5-1.3 |
|  | Homozygote |  | 24.2 | 0.8 | 0.5-1.3 | 0.8 | 0.5-1.4 |
|  | Reference |  | 24.8 | Ref |  | Ref |  |
| *TYMS* | Heterozygote | rs11540152 | 0.0 | - |  | - |  |
|  | Homozygote |  | 0.0 | - |  | - |  |
|  | Reference |  | 95.8 | Ref |  | Ref |  |
| *TYMS* | Heterozygote | rs2853532 | 43.7 | 0.9 | 0.6-1.2 | 1.0 | 0.6-1.5 |
|  | Homozygote |  | 16.4 | 0.6 | 0.4-1.0 | 0.9 | 0.5-1.5 |
|  | Reference |  | 36.2 | Ref |  | Ref |  |
| *TYMS* | Heterozygote | rs2847149 | 47.1 | **1.9** | 1.2-2.9 | 1.4 | 0.9-2.1 |
|  | Homozygote |  | 22.0 | **2.2** | 1.4-3.5 | 1.4 | 0.8-2.4 |
|  | Reference |  | 28.1 | Ref |  | Ref |  |
| *TYMS* | Heterozygote | rs1001761 | 48.8 | **1.8** | 1.2-2.7 | 1.3 | 0.8-2.0 |
|  | Homozygote |  | 21.5 | **2.4** | 1.5-3.8 | 1.5 | 0.9-2.5 |
|  | Reference |  | 28.7 | Ref |  | Ref |  |
| *TYMS* | Heterozygote | rs502396 | 50.7 | 1.5 | 1.0-2.3 | 0.9 | 0.6-1.5 |
|  | Homozygote |  | 18.9 | **2.1** | 1.3-3.3 | 1.3 | 0.7-2.2 |
|  | Reference |  | 28.1 | Ref |  | Ref |  |

Abbreviations: see footnote of Table 1.

^1^Frequency is given among the 359 controls used for comparison for spina bifida. Totals may not equal 100 owing to rounding or missing data.
